# Selective Elimination and Rationalization of Cell-based Assays in Deceased Donor Kidney Transplant Crossmatching

**DOI:** 10.1097/TXD.0000000000001603

**Published:** 2024-03-07

**Authors:** Myriam Khalili, Olusegun Famure, Michelle Minkovich, Kathryn J. Tinckam, Sang Joseph Kim

**Affiliations:** 1 Ajmera Transplant Centre, Toronto General Hospital, University Health Network, Toronto, ON, Canada.; 2 Division of Nephrology, University Health Network, University of Toronto, Toronto, ON, Canada.

## Abstract

**Background.:**

While there is increasing reliance on a negative virtual crossmatch to proceed with deceased donor kidney transplantation, a flow cytometry crossmatch (FCXM) is still usually performed after the transplant has already occurred. Our center has eliminated pretransplant physical crossmatches for most patients, and since 2018, we have eliminated the systematic performance of posttransplant FCXMs.

**Methods.:**

We studied all deceased donor kidney transplants in our program between June 1, 2018, and March 31, 2021, to evaluate the impact of eliminating retrospective FCXMs on resource utilization and graft outcomes (ie, the occurrence of antibody-mediated rejection [AMR] in the first 3-mo posttransplant).

**Results.:**

A total of 358 kidney transplants occurred during the study period, and approximately 70% of these transplants proceeded without the performance of any FCXM. Incidence rates of AMR were low (9.63 per 1000 person-months), which compared favorably with the incidence rate of AMR during the 3-y period preceding the policy (4.82 per 1000 person-months, *P* = 0.21).

**Conclusions.:**

Our results suggest that moving away from retrospective FCXM and relying exclusively on the virtual crossmatch is safe and efficient for kidney allocation.

## Graphical abstract

**Figure:**
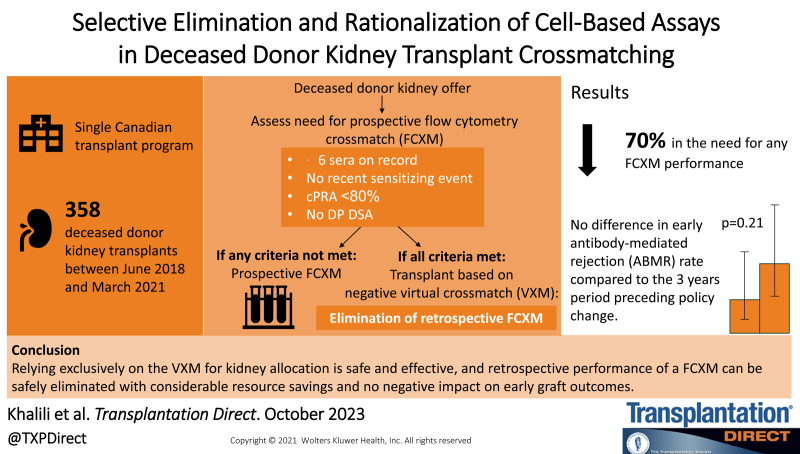

Crossmatching in the context of kidney transplantation relies on the identification of circulating antibodies directed against a potential donor’s HLA and is aimed at preventing hyperacute rejection episodes. The complement-dependent cytotoxicity assay was the first crossmatching method introduced and was clearly shown to be associated with high rates of hyperacute rejection if positive pretransplant.^1^ However, the poor sensitivity of complement-dependent cytotoxicity assays has paved the way for the development of more precise crossmatching methods, including the flow cytometry crossmatch (FCXM).^2^ More recently, the virtual crossmatch (VXM) has gained widespread use. VXM uses solid-phase assays such as single-antigen beads (SABs) to determine the HLA-antibody specificities of kidney transplant candidates and identify unacceptable antigens. This method accurately predicts the results of physical crossmatching,^3,4^ which improves the efficiency of the process.^5,6^ An increasing number of transplant programs are abandoning prospective cell-based crossmatches, relying solely on a negative VXM with favorable transplant outcomes.^7-9^ However, most centers that have published their experience on the safe omission of pretransplant physical crossmatch continue to systematically perform a retrospective FCXM, usually on the next working day after the transplant has been done.

In the province of Ontario, Canada, the pretransplant physical crossmatch was abandoned in 2014 and the provincial algorithm, managed by Trillium Gift of Life Network, has since used cumulative VXM as the pretransplant final crossmatch guiding allocation decision for most patients. Only under specific circumstances (ie, when there were an insufficient number of historical samples to map out an HLA trajectory) were stat cell-based crossmatches performed prospectively. In addition to this practice, the University Health Network’s kidney transplant program performed a FCXM on the immediate pretransplant (IP) serum the day after their transplant for all patients. However, given the improved sensitivity and specificity of solid-phase assays when compared with FCXM^10-13^ and that posttransplant FCXM did not impact the decision to proceed with transplantation, our center has eliminated the systematic performance of a retrospective FCXM since 2018.

The main objective of this study was to analyze kidney transplant outcomes since this change in policy to assess the feasibility and safety of omitting systematic performance of retrospective cell-based crossmatches, with our primary outcome of interest being biopsy-proven antibody-mediated rejection (AMR) incidence rates at 3-mo posttransplant. We hypothesized that retrospective cell-based crossmatches do not impact transplant risk assessment and decision making beyond the information derived from the SAB and that they can be safely abandoned with no unfavorable effect on graft outcomes and improved efficiency of the transplant process.

## MATERIALS AND METHODS

### Study Design and Data Collection

We performed a retrospective cohort study including all consecutive kidney transplants from deceased donors performed at the University Health Network between June 1, 2018, and March 31, 2021. Multiorgan transplants were excluded from this study, but patients with a previous transplant were not. Patients with no follow-up data up to 3 mo after their transplant date were also excluded.

The Comprehensive Renal Transplant Research Information System (CoReTRIS) was used to collect clinical data collection from kidney transplant recipients. CoReTRIS is an in-center clinical research database that includes demographic, clinical, laboratory, and diagnostic data of all patients and their donors followed by the University Health Network kidney transplant program since January 1, 2000. Further details on this database have been presented elsewhere.^14^ Donor data were collected from CoReTRIS and the Trillium Gift of Life Network. The hospital’s electronic patient records were used to access reports from the histocompatibility laboratory, which included information on panel reactive antibody (PRA), VXM, and FCXM results. This system was also used to collect pathology reports of allograft biopsies. Reasons motivating the need for a FCXM were inferred from the information available in the HLA reports. HLA mismatches with the donor were captured at the antigen level for the following loci: A, B, C, DRB1, DRB345, DQA, DQB, DPA, and DPB.

The University Health Network’s Quality Improvement Review Committee approved this study, and all participants provided informed consent before enrollment to participate in data collection for research purposes in the CoReTRIS database. This study adhered to the Declaration of Helsinki and its amendments.

### Antibody Screening, Crossmatching, and HLA Typing

Serum samples from waitlisted patients were sent to the HLA laboratory every 3 mo and screened for anti-HLA antibodies using LABScreen Single Antigen assays from One lambda. At the time of organ allocation, a cumulative VXM was performed, with all antibodies detected at any time point during the waiting period, and kidneys from deceased donors were offered based on a negative cumulative VXM, which was defined as the absence of donor-specific antibodies (DSAs) on all of the available serum samples. As such, DSA were not crossed. HLA typing for recipients has transitioned from polymerase chain reaction-reverse sequence specific oligonucleotides to next-generation sequencing (NGS) in June 2020. For donors, typing is initially performed with polymerase chain reaction-sequence-specific primer and since June 2020, NGS is retrospectively performed.

In 2014, a specific set of criteria was established to guide the safe omission of pretransplant physical crossmatches:

1. Presence of >6 sera on record, tested on solid phase assays
2. Absence of interval sensitizing event since the last tested serum on record
3. Calculated PRA <80%
4. Absence of indeterminate antibody specificities or DSA against DP antigens

If any of these criteria is not met, a stat FCXM using donor T and B cells is performed pretransplant using an IP sample (obtained at the time of patient admission) and historical serum(s). The results of this FCXM guides the decision to proceed with transplantation. If all criteria are met, transplantation proceeds with a negative VXM as the final crossmatch and testing of IP serum on SAB occurs within 1 working day. Before June 2018, a retrospective FCXM was still systematically performed. Since June 2018, no systematic FCXM was performed retrospectively but donor cells are kept for 96 h in case that one is needed.

### Outcomes

The primary outcome of interest was the incidence of AMR in the initial 3-mo period after transplant. Our center does not perform protocol biopsies. For each patient included in the study cohort, review of all for-cause allograft biopsy reports performed in the first 3 mo after transplant was undertaken, excluding donor time zero (implantation) biopsies. When >1 biopsy was performed during this predefined period, each one was analyzed separately and reported. Episodes of biopsy-proven histopathologic features of AMR were identified using the first 2 criteria for AMR from Banff’s 2019 meeting.^15^ For comparison, biopsy-proven AMR episodes in the first 3-mo posttransplant period were reviewed for the 3 y before the study period (June 1, 2015, to May 31, 2018). Any SAB testing performed in the first 3-mo posttransplant period to determine the presence of DSA was also evaluated.

### Statistical Analyses

For kidney transplant recipients and donors’ characteristics, continuous variables are presented as mean ± SD or median (interquartile range), depending on their distribution. The incidence rate of AMR in the 3-mo posttransplant (per 1000 person-months) for the 3-y study period was compared with the AMR rate in the 3-y period preceding the change in crossmatching policy using the mid *P*-value approach.^16^

## RESULTS

### Study Cohort

From June 1, 2018, to March 31, 2021, 358 deceased donor kidney-only transplants were performed at our center. Baseline recipient, donor, and transplant characteristics are presented in Table 1. The mean age at transplant was 56 y (± 12.8 y), and almost all transplant recipients (97%) received induction immunosuppression, the majority (71.2%) with T-cell depleting antibodies. As for the sensitization profile of our cohort, 57.5% of recipients were unsensitized to class I HLA antigens and 73.5% were unsensitized to class II antigens. The median HLA mismatch sum for class I loci was 5/6 and the median HLA mismatch sum for class II loci was 6/12. Some data were missing for the following HLA mismatches: DRB345 (n = 33), DQA (n = 1), and DPA/DPB (n = 1).

**TABLE 1.** Baseline characteristics of the study cohort (n_transplants_ = 358)

Variable	Characteristics
Recipient characteristics	
Age at time of transplant (y ± SD)	56.1 (± 12.8)
Female sex, n (%)	149 (41.6)
Race, AA, n (%)	33 (9.2)
Time on dialysis (mo), median (IQR)	60.1 (41.7–85.4)
Cause of ESRD	
Diabetes, n (%)	85 (23.7)
Glomerulonephritis, n (%)	113 (31.6)
Hypertension, n (%)	28 (7.8)
Polycystic kidney disease, n (%)	37 (10.3)
Other, n (%)	95 (26.5)
Donor characteristics	
Donor age (y)	46.4 (± 13.8)
Female sex, n (%)	120 (33.5)
Donor type	
DBD, n (%)	248 (69.3)
DCD, n (%)	110 (30.7)
Cold ischemia time (h)	9.2 (6.8–12.6)
DGF^*a*^, n (%)	122 (34.1)
Immunosuppression	
Induction therapy	
T-cell depleting antibody, n (%)	255 (71.2)
IL-2 receptor blocker, n (%)	92 (25.7)
No induction, n (%)	11 (3.1)
Maintenance IS	
CNI, n (%)	355 (99.2)
Antimetabolite, n (%)	357 (100)
mTORi, n (%)	7 (2.0)
Steroids, n (%)	354 (98.9)
Sensitization	
PRA^*b*^	
Class I, median (IQR)	0 (0–19)
Nonsensitized (PRA = 0%), n (%)	206 (57.5)
Sensitized	
1%–79%, n (%)	133 (37.2)
≥80%, n (%)	19 (5.3)
Class II, median (IQR)	0 (0–4)
Nonsensitized (PRA = 0%), n (%)	263 (73.5)
Sensitized	
1%–79%, n (%)	72 (20.1)
≥80%, n (%)	23 (6.4)
Kidney tx number	
1, n (%)	325 (90.8)
2, n (%)	31 (8.7)
>2, n (%)	2 (0.6)
Previous transplant (nonkidney), n (%)	14 (3.9)
HLA mismatches	
Class I, median (IQR)	5 (4–5)
Class II, median (IQR)	6 (4–7)

^*a*^ Dialysis in the first week posttransplant.

^*b*^ Class I and class II PRA from the latest sera before the kidney offer.

AA, African American; CNI, calcineurin inhibitors; DBD, donation after brain death; DCD, donation after circulatory death; DGF, delayed graft function; ESRD, end-stage renal disease; IL-2, interleukin-2; IQR, interquartile range; IS, immunosuppression; mTORi, mTOR inhibitors; PRA, panel reactive antibody; tx, transplant.

**FIGURE 1.**
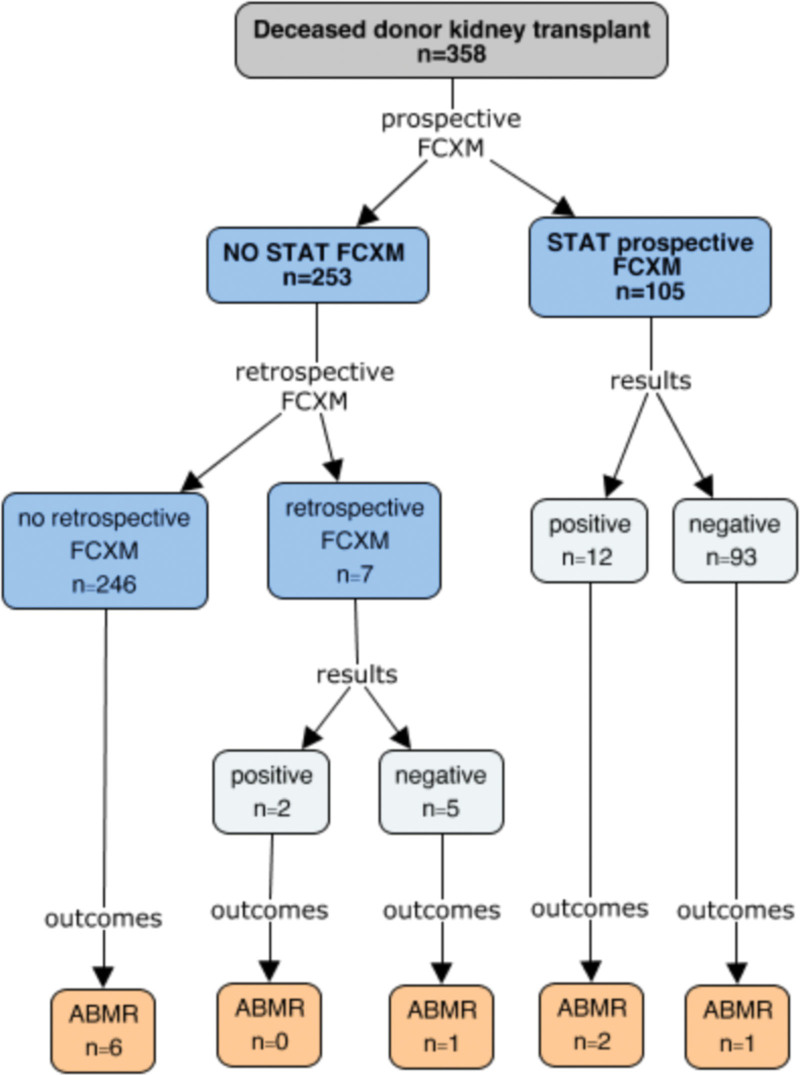
Flow diagram of FCXM for the study cohort (n = 358). AMR, antibody-mediated rejection; FCXM, flow cytometry crossmatch.

During the study period, 139 waitlisted patients underwent a prospective (stat) FCXM after receiving a deceased donor kidney transplant offer. Among these, there were 3 canceled transplants (2.2%) because of a positive FCXM result. In 22.3% of these cases (n = 31), the FCXM was negative, but the transplant was canceled due to non-HLA-related reasons. Thus, 75.5% (n = 105) of the waitlisted patients for whom a prospective FCXM was performed went ahead with transplant. An additional 253 patients were transplanted based on a negative cumulative VXM as the final crossmatch. For only 2.8% (n = 7) of these patients, a FCXM was performed retrospectively. Figure 1 depicts all physical crossmatches performed for the 358 deceased-donor kidney transplant recipients during the study period.

### Kidney Transplant Recipients With a Prospective FCXM (n = 105)

Among the 105 patients who were transplanted with a pretransplant FCXM, 11.4% (n = 12) had a positive result (Table 2). In all 12 cases, the absence of DSA was confirmed with the IP serum. These positive FCXM were alloreactive with B cells only in 3 cases, with T cells only in 4 cases, and both with T and B cells in 5 cases. Among these, 4 had a positive auto-crossmatch result. Two patients with an alloreactive FCXM experienced AMR episodes. Thirteen patients had missing auto-crossmatches, but none of these had alloreactive FCXMs. One patient had an unexpected positive result on the IP SAB testing; however, the FCXM was negative, and this patient did not experience early rejection.

**TABLE 2.** Results of crossmatch testing in transplanted patient (n = 358)

Patients with prospective FCXM (n = 105)		
Results	IP VXM negative	IP VXM positive
Negative, n	92	1
Positive attributed to HLA antibodies, n	0	0
Positive attributed to auto antibodies/non-HLA antibodies, n	12	0
Patients with VXM as the final pretransplant XM (n = 253)		
Results	IP VXM negative	IP VXM positive
No retrospective FCXM, n	246	0
Retrospective FCXM, n	6	1
Negative, n	4	1
Positive attributed to HLA antibodies, n	0	0
Positive attributed to auto antibodies/non-HLA antibodies, n	2	0

FCXM, flow cytometry crossmatch; IP, immediate pretransplant; VXM, virtual crossmatch; XM, crossmatch.

Reasons for performing a FCXM were not mutually exclusive. A total of 66 of these prospective cell-based crossmatches were performed because there were 6 serum samples or less screened for the presence of antibodies at the time of the organ offer. For 51 highly sensitized patients, calculated PRA justified the need for a pretransplant FCXM. An additional 2 FCXM were ordered because of the possibility of DSA on previous SAB test, and for 8 patients, the rationale for the prospective FCXM remained unclear from analysis of the HLA report. We could not capture cases for which a FCXM would have been performed because of a recent sensitizing event, as this is not clearly specified on HLA reports.

### Kidney Transplant Recipients With a Retrospective FCXM (n = 7)

A total of 2.8% (n = 7) of the recipients who underwent transplantation with a VXM as the final crossmatch had a retrospective FCXM performed after transplant. For 2 patients, posttransplant FCXM was performed because of a possible unexpected DSA with the inpatient VXM on arrival for transplant. In one case, further revision and testing of the SAB result confirmed that the IP VXM was in fact negative, whereas in the other, the FCXM was negative. In one case, the FCXM was planned to be performed prospectively due to a low number of sera but due to an insufficient number of donor cells, the FCXM was performed retrospectively upon arrival of new donor sample. In the other 4 cases, the reasons were unclear.

Among the 7 kidney transplant recipients with retrospective FCXMs, 2 had a positive B-cell flow crossmatch result, one of which also had a positive B-cell auto-crossmatch, and none had positive T-cell crossmatches (Table 2). These 2 FCXM were interpreted as immunologically irrelevant, and no AMR episodes occurred in these patients. Three of the 7 patients had missing auto-crossmatches, but all had negative allo-B- and T-cell FCXM.

### Biopsy Results (n = 105)

For cause biopsies in the first 3-mo posttransplant were performed in 23.7% (n = 85) of the cohort. Since some patients had >1 biopsy performed during this early period, a total of 105 biopsy reports were available for analysis (**Table S1** [**SDC**, http://links.lww.com/TXD/A632]). There were 10 episodes of early histopathologic features of AMR (Table 3). All patients had active AMR, except patient number 3, who had biopsy features of chronic AMR with early transplant glomerulopathy at 60 d posttransplant. A total of 14 biopsies meeting criteria for AMR were noted in these 10 patients, and the majority (78.6%) were negative for C4d staining. A total of 14.3% of biopsies (n = 2) showed signs of concomitant T-cell–mediated rejection grade IIA (patient numbers 5 and 10). A third patient had biopsy features meeting Banff criteria for borderline for acute T-cell–mediated rejection (patient number 7). DSA were discovered at the time of the biopsy in 3 patients, and all were class II DSA.

**TABLE 3.** AMR episodes (n = 10)

Patients	Biopsy number	Time posttransplant (d)	PRA^*a*^	C4d positivity	Concomitant histologic diagnostic	VXM^*b*^	FCXM	Posttransplant DSA
1	1	14	0/28	Y	ATN	−	Y (retrospective): negative	Y
	2	26		Y	N			
2	1	9	0/0	N	N	−	N	Y
3	1	60	0/0	N	ATN	−	N	N
4	1	8	0/0	N	ATN	−	N	N
5	1	8	0/0	N	TCMR (IIA)	−	N	N
6	1	8	0/0	Y	N	−	Y (prospective): positive^*c*^	N
	2	69		N	N			
7	1	15	0/0	N	N	−	N	N
8	1	67	90/98	N	ATN	−	Y (prospective): positive^*d*^	N
9	1	10	0/0	N	N	−	Y (prospective): negative	N
10	1	16	0/0	N	N	−	N	Y
	2	30		N	N			
	3	46		N	TCMR IIA			

^*a*^ % class I/% class II represents the PRA for class I and class II with the inpatient sera.

^*b*^ VXM result at time of transplant with the IP serum; −: negative result; +: positive result.

^*c*^ Positive FCXM: alloreactive with B and T cells (IP and historic sera). Standard immunologic risk.

^*d*^ Positive FCXM: alloreactive with B and T cells (IP and historic sera) and autoreactive with B and T cells. Standard immunologic risk.

AMR, antibody-mediated rejection; ATN, acute tubular necrosis; DSA, donor-specific antibody; FCXM, flow cytometry crossmatch; IP, immediate pretransplant; N, no; PRA, panel reactive antibody; TCMR, T-cell–mediated rejection; VXM, virtual crossmatch; Y, yes.

All patients presenting with early AMR episodes had a negative historical cumulative VXM as well as negative SAB at the time of arrival for transplant. Four patients underwent FCXM: 3 prospectively and 1 retrospectively. In 2 cases, the prospective FCXM was B-cell and T-cell positive. For patient number 6, FCXM was performed because there were not enough serum samples and since there were no DSA with SAB testing, the positive FCXM was attributed to non-HLA antibodies and the patient was deemed at standard immunologic risk. The patient did not develop any DSA posttransplant. The second patient (patient number 8) was highly sensitized (pretransplant class I PRA 90%/class II PRA 98%) and received a second kidney transplant from a zero HLA-mismatched donor. The patient had delayed graft function, with 2 allograft biopsies at 9- and 17-d posttransplant showing no signs of AMR. Finally, a third biopsy 67 days posttransplant showed microvascular inflammation (Banff score glomerulitis, peritubular capillaritis) without positive staining for C4d and continued absence of identified DSA.

**FIGURE 2.**
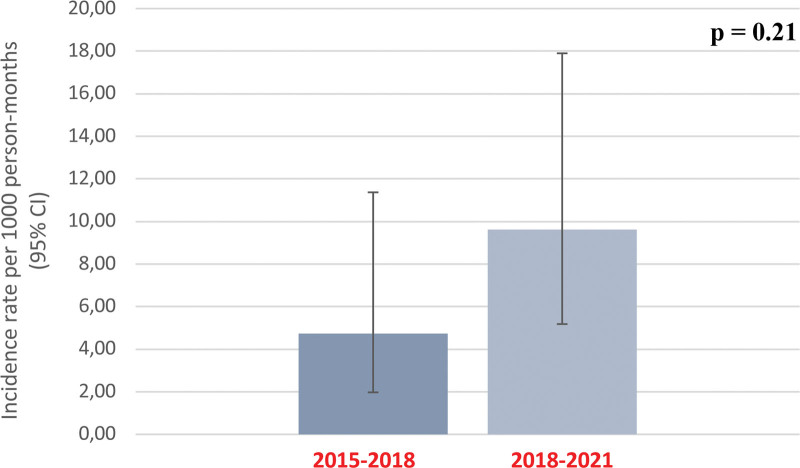
Comparison of early posttransplant AMR rates before and after elimination of systematic performance of retrospective FCXM. AMR, antibody-mediated rejection; FCXM, flow cytometry crossmatch; CI, confidence interval.

The incidence rate of acute AMR in the early posttransplant period during the study period was of 9.63 episodes per 1000 person-months (95% confidence interval, 5.18-17.90). We compared this to the AMR incidence rate during the 3-y period before the 2018 change in policy (June 1, 2015, to May 31, 2018), during which 353 deceased donor kidney-only transplants were performed. Baseline characteristics of this historical cohort are provided in **Table S2 (SDC**, http://links.lww.com/TXD/A632). During that period, a total of 5 episodes of biopsy-proven AMR in the first 3-mo posttransplant were identified. The AMR incidence rate during that period was 4.82 events per 1000 person-months (95% confidence interval, 2.00-11.57), which was not significantly different from the study period AMR incidence rate (*P* = 0.21), as shown in Figure 2.

### Cost Savings With the Elimination of Systematic Retrospective Posttransplant FCXM

During the study period, 68.7% (n = 246) of all deceased donor kidney transplants proceeded without a FCXM, whether pretransplant or posttransplant. Performance of a standard FCXM in our center is CAN$754.24, which include cost for the test and technician time. When a stat FCXM is performed outside of working hours, which occurs in about 75% of cases, there are additional costs of $420, mainly due to overtime for laboratory technicians. During our study period, 139 stat FCXM and 7 retrospective FCXM were performed, with an estimated total cost of CAN$153904.04. We estimated a cost savings of CAN$185543.04 during the study period by avoiding retrospective crossmatches among 246 kidney transplant recipients.

## DISCUSSION

This single-center retrospective study explored the feasibility and safety of eliminating the systematic use of posttransplant (retrospective) FCXM in the context of deceased donor kidney transplantation. During the 34-mo period of this study, nearly 70% (n = 246) of all deceased donor kidney transplants proceeded without a prospective or retrospective physical crossmatch, which was highly cost- and resource-effective and did not unfavorably impact graft and patient outcomes. Indeed, we demonstrated that the incidence rate of early histopathologic features of AMR remained low in our program (9.63 episodes per 1000 person-months), with no significant difference when compared with the 3-y period before the change in policy, confirming its safety. The AMR rate at 3-mo posttransplant was chosen as the primary outcome because any crossmatch technique performed at the time of transplant will aim to capture the risk of AMR from the identification of preformed DSA. Later onset AMR episodes are usually caused by de novo DSA, which cannot be predicted by antibody screening at the time of transplant. If performance of retrospective FCXMs was adding useful information about low-level DSA not captured by a pretransplant VXM, we could hypothesize that, as the result is obtained within 1–2 d after transplant, it could still inform decisions around immunosuppression protocols (eg, extending antilymphocyte therapy or initiating more aggressive antibody-directed treatments) which would modify the early AMR risk. However, we were able to demonstrate that, as we had hypothesized, there is no negative impact of the omission of cell-based crossmatches after transplant on the rate of early AMR. We also found that even when the use of prospective FCXM is restricted to a small subset of patients at higher risk, these very rarely yielded unexpected positive results when the VXM was negative with only 2.2% of the pretransplant FCXM performed during the study period being unexpectedly positive with results deemed immunologically relevant and leading to the transplant being canceled.

With the evolution and refinement of solid-phase assays in the last decades, any potential added value of cell-based crossmatch techniques such as the FCXM in kidney transplantation has become uncertain. Multiple groups have compared solid-phase immunoassays to physical crossmatches such as the FCXM in their prognostic capacity regarding graft outcomes and have shown better sensitivity and specificity of solid-phase assays. These studies have demonstrated that a negative VXM, even in the presence of a positive FCXM, is associated with a favorable prognosis. False positive FCXM results, which occur in up to 20% of transplanted patients in published studies,^12^ are usually directed against non-HLA antigens^17^ or represent nonspecific binding of therapeutic antibodies to the Fc receptors of donor lymphocytes (eg, rituximab). On the other hand, a positive VXM can identify subgroups at higher risk for AMR despite a negative FCXM result^10-13^ and data on the relevance of DSA in the presence of negative FCXM increasingly seems to favor an association with AMR.^18,19^ Thus, despite the limitations of the technique,^20^ solid-phase assays display improved capacity for identification of unacceptable antigens and have led to an increasing number of transplant programs abandoning the systematic use of prospective cell-based crossmatches.^7-9^

In the United States, 19% of transplants in 2018 proceeded with the omission of a physical crossmatch, compared with 9% before the 2014 change in allocation policy, leading to a significant reduction in cold ischemia time and no safety signals.^9^ Even in highly sensitized patients, Roll et al^21^ showed that omission of a pretransplant physical crossmatch is safe. These changes in policies regarding the use of VXM as the final pretransplant crossmatch are in line with the most recent histocompatibility guidelines. As such, the American Society for Histocompatibility and Immunogenetics’^22^ states that laboratories should perform crossmatching using an assay with sensitivity appropriate for the protocols established with the transplant center (D.5.3.2.1.3). Similarly, the Transplantation Society^23^ and British Transplantation Society^24^ recommend sensitive antibody identification, which can be used in a VXM as the final prospective crossmatch, given that certain criteria are met.

Yet, while an increasing number of transplant programs adopt policies that abandon the necessity for a negative cell-based crossmatch result before proceeding with transplantation, they still perform a FCXM retrospectively for all patients. However, physical crossmatching should not be viewed as a reliable strategy to overcome the limitations of solid-phase assays. With improvements in antibody detection and typing techniques, we argue that there is no added value to either a prospective or retrospective physical crossmatch, which takes an average of 3–4 h to perform by a dedicated laboratory technician. In their 2021 guidelines, the BTS, in collaboration with the British Society for Histocompatibility and Immunogenetics, stated as a grade 2B recommendation that a retrospective cell-based crossmatch may be safely omitted when a pretransplant VXM has been used “if an audit of sufficient cases shows concordance between the initial VXMs and subsequent leucocyte XMs.”^25^

The identification of unacceptable antigens is a complex task that demands integration and critical analysis of HLA laboratory testing results with the clinical context of a given patient, and collaboration between the HLA laboratory team and transplant clinicians is of utmost importance. The reliability of VXM and correct identification of unacceptable antigens are highly dependent on comprehensive and high-resolution molecular HLA typing of donors at all HLA loci. However, the implementation of a similar approach in other programs requires increased use of high-resolution donor-typing techniques, such as NGS, for reliable comparison with a recipient’s identified HLA antibodies.

To our knowledge, this is the first study relaying the experience of a large volume transplant center on the complete elimination of cell-based crossmatches, except for higher immunologic risk patients. Limitations of our study include the retrospective single-center design and the relatively short study follow-up period since the 2018 policy change. Accordingly, there were a small number of primary outcome events, which prevented the performance of multivariable analysis to identify factors independently associated with early rejection episodes, as the power was insufficient for such analyses. Another limitation is the lack of a direct control group. Instead, we compared outcomes from the first 3-y period since the change in policy with outcomes observed in the 3-y period before the change in policy. However, the current and historical cohorts were very similar as there was no other practice change during that period. Also, testing for the presence of non-HLA antibodies in cases of DSA-negative AMR episodes was not performed. Potential role of non-HLA antibodies such as antibodies directed against MHC class I-related chain A antigen, or the angiotensin II type 1 receptor in the pathogenesis of a subset of AMR episodes is of great interest.^26,27^ However, assays to assess for the presence of non-HLA antibodies are not as validated as for anti-HLA antibodies, and non-HLA typing is not a standard practice, which limits the interpretation of antibody results. Finally, the cost analysis presented in this study is not a comprehensive cost analysis and represents an estimate of the direct overall cost savings of abandoning the systematic performance of retrospective FCXMs.

Refinement of solid-phase assays for antibody identification and HLA typing technology is increasingly leading to reliance on VXM to proceed with kidney transplant for most patients, with demonstrated safety. We now show that the retrospective performance of a FCXM has no added value and can be safely eliminated with considerable resource savings and no negative impact on early graft outcomes.

## ACKNOWLEDGMENTS

We thank the students of the Multi-Organ Transplant Student Research Training Program (MOTSRTP) for their efforts in collecting, entering, and auditing data in CoReTRIS.

## Supplementary Material


