# Supplementary material for: Selective Elimination and Rationalization of Cell-based Assays in Deceased Donor Kidney Transplant Crossmatching
Source: Transplant Direct. 2024 Mar 7;10(4):e1603. doi: 10.1097/TXD.0000000000001603 (PMC10923350; doi:10.1097/TXD.0000000000001603)
Supplement: Supplementary file 1 [file txd-10-e1603-s001.pdf]

Table S1. Description of for-cause biopsy results in the first 3 months post-transplant

Characteristics	Result, n (%)
**Number of biopsies per patient (n=358)**	
0	273 (76.3)
1	69 (19.3)
2	12 (3.4)
3	4 (1.1)
**Biopsy results[a] (n=105)**	
**ABMR**	14 (13.3)
**Acute TCMR or borderline for acute TCMR**	17 (16.2)
• borderline for acute TCMR	8 (47.1)
• TCMR grade IA	2 (11.8)
• TCMR grade IB	3 (17.6)
• TCMR grade IIA	4 (23.5)
**ATN**	80 (76.2)
**Glomerular disease**	6 (5.7)
• Thin basement membrane disease	2 (33.3)
• Recurrence of primary PLA2R positive MN	1 (16.7)
• Recurrence of FSGS	1 (16.7)
• Fibrillary glomerulonephritis	1 (16.7)
• BK nephropathy	1 (16.7)
**No gross abnormality**	7 (6.7)

[a]Each biopsy could hold more than one diagnostic

ABMR, antibody mediated rejection; TCMR, T cell mediated rejection; ATN, acute tubular necrosis; MN, membranous nephropathy

Table S2. Baseline characteristics of transplants from June 2015 to May 2018 ($n_{transplants}$= 353)

Variable	Characteristics
**Recipient characteristics**	
Age at time of transplant (years± SD)	56.8 (± 11.9)
Female sex, n (%)	131 (37.1%)
Race, AA, n (%)	22 (6.2%)
Time on dialysis (months)	57.5 (34.1, 78.7)
Cause of ESRD	
Diabetes, n (%)	108 (30.8%)
Glomerulonephritis, n (%)	127 (36.2%)
Hypertension, n (%)	19 (5.4%)
Polycystic kidney disease, n (%)	28 (8.0%)
Other, n (%)	69 (19.7%)
**Donor characteristics**	
Donor age (years)	49.4 (± 16.5)
Female sex, n (%)	147 (41.6%)
Donor type	
DBD, n (%)	247 (70.0%)
DCD, n (%)	104 (29.5%)
Cold ischemia time (hours)	9.4 (6.8, 12.5)
DGF[a] , n (%)	133 (37.7%)
**Immunosuppression**	
**Induction therapy**	
T cell depleting antibody, n (%)	284 (80.5%)
IL-2 receptor blocker, n (%)	63 (17.9%)
No induction, n (%)	6 (1.7%)
**Sensitization**	
**PRA[b]**	
Class I: median (IQR)	0 (0, 4)
Nonsensitized (PRA=0%): n (%)	214 (70.6%)
Sensitized:	
1% - 79%: n (%)	79 (26.1%)
≥ 80%: n (%)	10 (3.3%)

Class II: median (IQR)	0 (0, 1)
Nonsensitized (PRA=0%): n (%)	228 (74.8%)
Sensitized:	
1% - 79%: n (%)	60 (19.7%)
≥ 80%: n (%)	17 (5.6%)
**Kidney tx number**	
1	319 (90.4%)
2	32 (9.1%)
>2	1 (0.3%)
**Previous transplant (non-kidney), n (%)**	9 (2.6%)

Legend:

[a] Dialysis in the 1st week post transplant

[b] Class I and class II PRA from the latest sera before the kidney offer.

DCD, donation after cardiac death; DBD, donation after brain death
